# Knowledge and attitudes to cardiopulmonary resuscitation (CPR)– a cross-sectional population survey in Sweden

**DOI:** 10.1016/j.resplu.2020.100071

**Published:** 2021-01-29

**Authors:** Cecilia Andréll, Camilla Christensson, Liselott Rehn, Hans Friberg, Josef Dankiewicz

**Affiliations:** a Center for Cardiac Arrest, Department of Clinical Sciences Lund, Faculty of Medicine, Lund University, Remissgatan 4, S-221 85 Lund, Sweden; b Practicum Clinical Skills Centre, Region Skåne. Barngatan 4, S-221 85 Lund, Sweden; c Department of Intensive and Perioperative Care, Skåne University Hospital, Carl-Bertil Laurells Gata 9, S-205 02 Malmö, Sweden; d Department of Cardiology, Skåne University Hospital, Entrégatan, S-221 85 Lund, Sweden

**Keywords:** Out-of-Hospital cardiac arrest, CPR, Training, Smartphone app responder, Laypeople, Attitudes

## Abstract

**Aim of the study:**

Rates of bystander CPR are increasing, yet mortality after out-of-hospital cardiac arrest (OHCA) remains high. The aim of this survey was to explore public knowledge and attitudes to CPR. Our hypotheses were that recent CPR training (< 5 years) would be associated with a high-quality response in a case vignette of OHCA with agonal breathing, and associated with an interest to become a smartphone app responder in suspected OHCA.

**Methods:**

Data were collected through a web survey. Respondents (≥18 years) in Skåne County, Sweden were members of a panel created by a market research company. Data were weighted with respect to gender, age, municipalities and level of education to increase generalisability to the general population.

**Results:**

A total of 1060 eligible answers were analysed. Seventy-six percent of non-healthcare professionals (n = 912) had participated in a CPR course at some time in life, 58 percent during the previous five years. The recommended CPR algorithm was known by 57 percent, whereas knowledge of the location of the nearest automated external defibrillator (AED) in a home environment was poor. Recent CPR training (< 5 years) did not favour high-quality response in a case vignette of OHCA with agonal breathing, but was one predictor of wanting to become a smartphone app responder.

**Conclusion:**

This study highlights possible areas of improvement in CPR training, which might improve OHCA identification and facilitate knowledge retention. The potential to recruit smartphone app responders seems promising in certain groups.

## Highlights

• Society knowledge of early interventions is rare in OHCAs with agonal breathing.
• CPR training should highlight methods improving retention of theoretical knowledge.
• The society may overestimate the OHCA survival to ‘television levels’.
• CPR training among the elder is a challenge requiring attention.
• Recruitment of smartphone app responders in suspected OHCA seems promising.

## Introduction

Out-of-hospital cardiac arrest (OHCA) is a time critical condition, in which citizens need to act quickly by making an emergency call and initiating bystander cardiopulmonary resuscitation (B-CPR).^1^

With longer ambulance response times,^2^ CPR by laypeople prior to ambulance arrival is increasingly important.^3^ The chance of survival is 50–70 percent higher if defibrillation is initiated within the first minutes.[4], [5] In order to reduce no-flow and low-flow times, first responder programs (fire fighters, police officers or smartphone app responders) have been implemented to a varying extent. Prehospital response schemes in suspected OHCA differ between and within countries. Some regions have a diverse combination of responder programs, while others have none.[6], [7], [8] The use of a smartphone app for laypeople response is one promising scheme that may increase B-CPR interventions.^8^

Mortality after OHCA remains high, approximately 90 percent.[9], [10] In order to increase survival, Deakin advocates increased focus on early links in the chain of survival, by improving OHCA recognition and B-CPR.^11^ European Resuscitation guidelines (2015) emphasise education of laypeople and recommend a curriculum including basic life support training focusing on recognition/identification of unconscious patients with no or abnormal breathing, and early CPR including good-quality chest compressions.^12^ Abnormal breathing (agonal breathing or ‘gasping’) occurs in approximately 40 percent of OHCA,^13^ and is a known confounding factor when identifying OHCA in emergency calls.^14^

For widespread B-CPR to become available, there needs to be a willingness to intervene.[15], [16], [17] Bakke et al. (2017) reported high numbers of first aid trained laypeople in Norway (90%) as well as positive attitudes towards performing CPR among respondents who had experienced a first aid situation.^18^ An association between CPR training and B-CPR is supported elsewhere.[19], [20] In contrast, a survey in Japan, with a low rate of CPR educated citizens (35%), showed low willingness to perform B-CPR, regardless of whether the person in cardiac arrest was a near relative (13%) or a stranger (7%).^21^ Brinkrolf et al. (2017) reported a low B-CPR rate (30%) in Germany,^22^ whereas another German study reported a 75 percent willingness to start B-CPR.^23^ Thus, knowledge gaps remain regarding knowledge and attitudes to CPR in society. Also, little is known about whether CPR-training is associated with an interest in becoming a smartphone app responder.

The aim of this survey was to assess CPR knowledge, experience, and willingness to act among Swedish citizens. We also studied how knowledge and attitudes were associated with a willingness to become a smartphone app responder. Our hypotheses were that recently CPR-trained citizens (< 5 years) would provide better responses to a case vignette of OHCA with agonal breathing, and that recently CPR-trained respondents would report a higher likelihood of becoming a smartphone app responder than non-trained ones.

## Methods

### Study design

This is an observational study based on a web survey by Ipsos, a market research company conducting market surveys, political opinion polls and research studies.^24^ Ipsos has a web panel of participants who have accepted to receive invitations to different kinds of studies through e-mail links. Ipsos uses a compensation scheme with intermittent payment. Data for this study was collected between July 8–16 2019, and rendered a compensation of SEK 14 (1.3 EUR). Data is presented in line with STROBE guidelines^25^ and good practice in reporting of survey research.^26^

### Setting and participants

Data were collected from the adult (≥18 years) population in the Skåne region (1.4 million inhabitants) The mean age in Skåne is 40.9 vs. 41.3 years in the general Swedish population, 50.1 percent (vs 49.7) are female, and 29.1 percent (vs. 28.5) have attended university.^27^ Skåne has a larger population density and a higher number of larger cities than the average of Sweden *(Table A1).*^28^

According to the study protocol, an adjustment for data bias through post-stratification was performed for increased generalisability. Data were weighted by Ipsos with respect to gender, age, municipalities and level of education.

### Quantitative variables

Questions about CPR training were based on the Utstein-style guidelines for OHCA.^29^ The agonal breathing case vignette, and CPR experience in general were derived or adapted from Bakke et al. (2017), including the addition of response alternatives for previously open-ended questions.^18^ Basic sociodemographic data, including a general health condition question from the Swedish public health survey,^30^ were added along with the smartphone app responder questions.

The questionnaire comprised 27 general questions and 14 follow-up questions. The questionnaire was divided into four parts: background variables, questions about knowledge and attitudes to CPR, an OHCA case vignette with associated questions, and questions regarding interest in becoming a smartphone app responder for suspected cardiac arrest. The questionnaire and sociodemographic data (*Table A2*) can be found in the *Supplementary Appendix*.

*Age groups* were defined in relation to ‘working age’ (i.e. 18−65 years vs. > 65 years). *Country of birth* was dichotomised to Nordic countries (Sweden, Denmark, Norway, Finland, Iceland) vs. other countries. *The classification of municipalities* follows the Swedish Association of Local Authorities and Regions^28^ (*Table A1*).

*Level of education* was based on a classification by Statistics Sweden^27^ and dichotomised to ‘no university education’ vs. ‘any university education.’ Data were divided in; *non-healthcare professionals* and *healthcare professionals.*

**Fig. 1:**
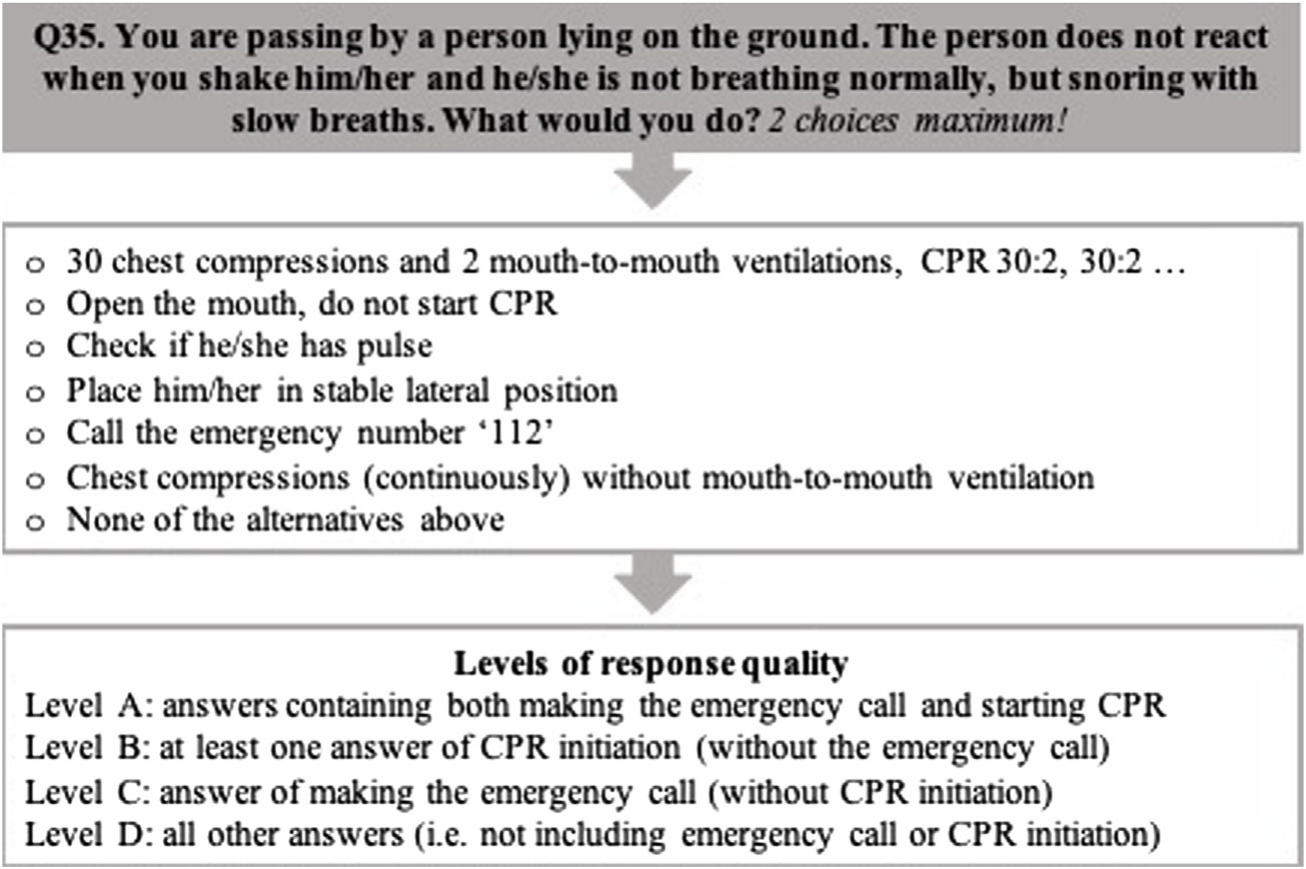
Cardiopulmonary resuscitation (CPR).

We defined *four levels of response quality* (Level A–D), based on seven response options in the case vignette with OHCA and agonal breathing (Fig. 1).

A Making an emergency call and starting CPR.
B Starting CPR (without an emergency call).
C Making an emergency call (without CPR initiation).
D All other answers (not including an emergency call or CPR initiation).

**Fig. 2:**
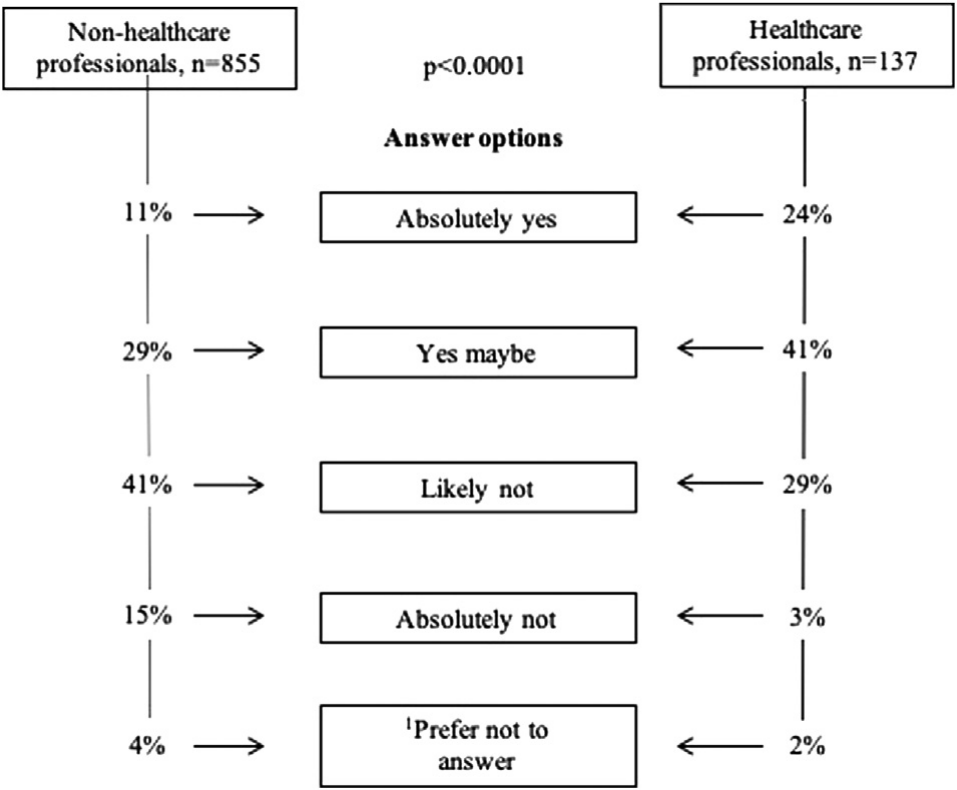
Weighted analysis by gender, age municipalities and level of education. Data on participants estimating their own health good enough to perform CPR (n = 992) stratified to non-healthcare professionals vs. healthcare professionals. Variables do not sum up to 100 percent as percentages are rounded off. ^1^’Prefer not to answer’ excluded in Linear-by-linear *X*^2^-Tests.

Options regarding the interest to become *a smartphone app responder* (Fig. 2) were ‘absolutely yes’, ‘yes maybe’, ‘likely not’, ‘absolutely not’, ‘prefer not to answer’, which was followed by a multiple choice question about the reason for their response, (*Supplementary Appendix)*.

The study was approved by the national Ethical Review Board (2019/02141). Data collection was based on active voluntary participation and informed consent was obtained, with non-mandatory answers for potentially sensitive questions, such as ethnicity, health status or monthly income.

### Statistical methods

Categorical data are presented as numbers and percentages, continuous data as median with first and third quartile and mean with standard deviation. Descriptive data (Table 1) is presented both unweighted and weighted. In the results section, unless stated otherwise, descriptive data is reported as weighted data. The logistic regression analyses were carried out using unweighted data. For univariable comparisons, Pearson’s *x*^2^-test was used for dichotomous data. The *x*^2^ Linear-by-linear test was used for the association between smartphone app responder willingness and health profession status (Fig. 2). Categorical variables (age group, country of birth, municipalities, level of education and healthcare profession) were dichotomised in the analyses.

**Table 1:** Baseline characteristics on study population.

		Study population n=1060 (%)	Weighted population^a^ (%)
Gender	Male	524 (49.4)	(49.5)
Age (years)	Median, q1-q3	53, 39−69	48, 33−67
	Mean, ±SD	53.3, 17.3	49.7, 18.4
Age groups (years)	18−65	724 (68.3)	(72.8)
	>65	336 (31.7)	(27.2)
Country of birth	Sweden or another Nordic country	1011 (95.4)	(95.2)
	All other countries	49 (4.6)	(4.8)
Municipalities	Large, medium-size or small cities	657 (62.0)	(54.2)
	Commuting or rural municipalities	403 (38.0)	(45.8)
Level of education	Any university education	653 (61.6)	(39.1)
	No university education	407 (38.4)	(60.9)
Occupation	Working	586 (55.3)	(57.0)
	Other^b^	474 (44.7)	(43.0)
Healthcare professions	Assistant nurse	76 (7.2)	(10.1)
	Nurse	55 (5.2)	(2.8)
	Physician	19 (1.8)	(1.1)
Long-term illness/	Yes	51 (4.8)	(5.2)
inconvenience or	No	998 (94.2)	(93.6)
disabilities that restrict	Prefer not to tell	11 (1.0)	(1.2)
CPR performance			

Cardiopulmonary resuscitation (CPR).

a Weighted analysis with respect to gender, age, municipalities and level of education. Percent (%). First and third quartile (q1-q3). Standard deviation (SD).

b Others (students, unemployed, sick leave or pensioner).

In order to avoid multicollinearity, a correlation matrix was carried out, with the cut off level set to Pearson r = 0.70 before the multivariable logistic regression analyses.^31^ Due to substantial correlation between age and occupation (r = 0.69), only age was used in the logistic regression analyses. SPSS version 26.0 (IBM Institute, Chicago, USA) was used. The significance level was defined as *p* value <0.05.

## Results

A total of 2904 web panel members were asked to participate; 1060 (37%) accepted and delivered valid answers (n = 3 excluded due to postal code outside Skåne County). The unweighted study population was equally divided between males and females. Fourteen percent (n = 150) reported having a healthcare profession. Age was higher than in the general population (Table 1), and 40 percent of respondents had previously called the emergency number 112 (unweighted data).

### Knowledge of cardiac arrest, CPR and AED among non-healthcare professionals

Twenty-six percent estimated the 30-day survival after OHCA to be 5–10 percent, 36 percent estimated it to be 30 percent and another 37 percent suggested 50 percent. Fifty-seven percent identified the recommended CPR algorithm (30:2) and 73 percent stated familiarity with the automated external defibrillator (AED) sign. Eighty-five percent estimated the survival to be 70 percent if an AED was used within three minutes. Regarding AED placement, 62 percent of students and people who work knew the location of the closest AED in school or at work, whereas 20 percent knew the location in their home environment.

### Experience of CPR training

Among non-healthcare professionals, 76 percent (n = 690) had attended a CPR course at some point in life. The median number of training sessions among respondents who had attended a CPR course was three. Fifty-eight percent (n = 399) had attended a CPR course within the last five years, of whom 29 percent (117/399) did so during the last year. Working age, female gender and university education were associated with CPR training during the last five years (Table 2). *Among healthcare professionals,* 95 percent (n = 141) had participated in a CPR course, 76 percent during the last five years (n = 107), 38 percent of whom (n = 41/107) during the last year.

**Table 2:** Attended a CPR course during the last five years among non-healthcare professionals.

	Logistic regression analysis	
	Univariable OR (95% CI)	Munivariable OR (95% CI)
Age 18−65 years^a^	4.93 (3.53−6.87)	4.91 (3.50−6.87)
Male gender	0.76 (0.58−0.99)	0.73 (0.55−0.97)
Born in a Nordic country^b^	0.82 (0.43−1.56)	0.99 (0.50−1.97)
Living in a city^c^	0.80 (0.61−1.05)	0.76 (0.57−1.01)
Any university education	1.43 (1.09−1.88)	1.35 (1.01−1.81)

Logistic regression analysis on unweighted data among non-healthcare professionals (n = 910), OR = Odds Ratio. CI = Confidence interval.

a 18−65 vs. > 65 years.

b Nordic country vs. all other countries.

c Cities vs. all other municipalities.

Providers of CPR training for non-healthcare professionals (n = 222 missing) were employers (49%), private companies (12%), voluntary organizations or others (10% each), vocational training (6%), elementary/high school (7%), military training (4%), or a driving license course (0.3%), 2 percent did not remember.

### Experience of cardiac arrest and CPR among non-healthcare professionals

**Fig. 3:**
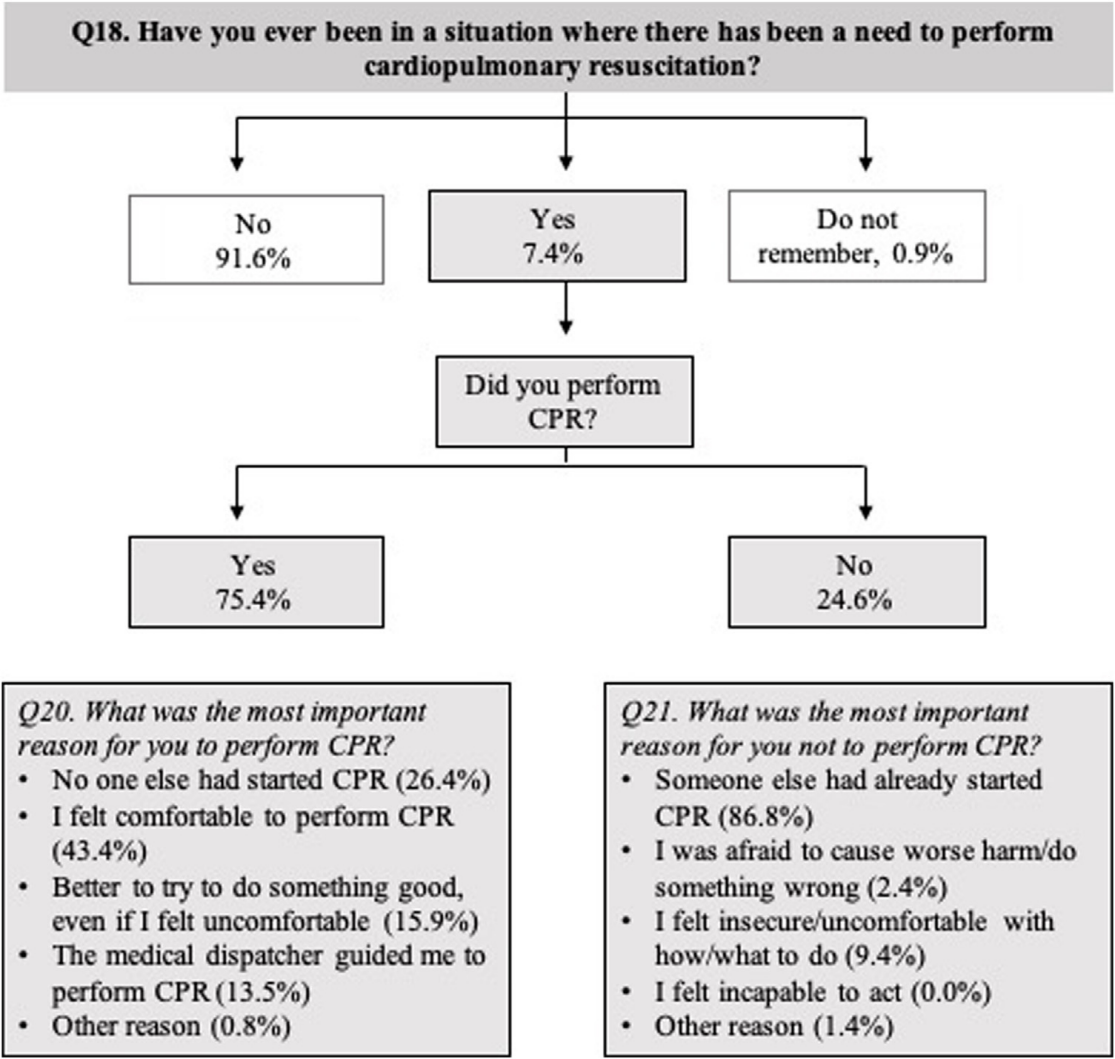
Weighted analysis by gender, age municipalities and level of education. Percent (%). Cardiopulmonary resuscitation (CPR).

Seven percent (n = 68) had experience of a CPR situation where 75 percent started CPR (Fig. 3). The most common intervention approach was ‘CPR 30:2’ (68%) followed by ‘chest compressions only’ (25%) and ‘ventilation only’ (7%). Reasons to start or not to start CPR are presented in Fig. 3. Among those who had CPR experience *after* CPR training, 93 percent completely or partly agreed that the CPR course made them more prepared for the situation.

### Case vignette - OHCA with agonal breathing

In the case vignette (Fig. 1), the most common answer among the non-healthcare professionals was to make an emergency call solely or in combination with other options (not including start of CPR, 71%), categorised as response quality Level C, in contrast to Level A (7%), B (6%) or D (16%, *Table A3)*. Having attended a CPR course during the last five years was not more common among Level A respondents (40%) than among Level B–D respondents (44%, p = 0.549). No significant predictors for a Level A response were identified, apart from being > 65 years (*Table A4*). Similar results were found if recent CPR training was defined as within 1 year (*Table A5).*

### Interest to become a smartphone app responder

Stratified data on non-healthcare professionals vs. healthcare professionals, (Fig. 2) showed an increased interest to become a smartphone app responder among healthcare professionals (p < 0.0001).

Among non-healthcare professionals who had participated in a recent CPR course (< 5 years), significantly more responded ‘absolutely yes’ or ‘yes maybe’ (56%), compared to ‘likely not’ or ‘absolutely not’ (39%), p < 0.0001. The association remained after controlling for other confounders (OR:1.60 [CI:1.19−2.16], Table 3). Working age and male gender were also associated with an interest to become a smartphone app responder. Main reasons among those responding positively were ‘a willingness to help people in need’ or ‘better to try than do nothing at all’, as compared to ‘lack of knowledge to perform CPR’ or ‘afraid of causing harm’ among those responding negatively.

**Table 3:** Factors associated with the answer ‘absolutely yes’ or ‘yes maybe’ to become a smartphone app responder in suspected cardiac arrest among non-healthcare professions with self-estimated health good enough to perform CPR.

	Logistic regression analysis	
	Non-healthcare professionals (n=861)	
	Univariable OR (95% CI)	Multivariable OR (95% CI)
Age 18−65 years^a^	3.31 (2.37−4.61)	2.76 (1.94−3.92)
Male gender	1.61 (1.22−2.12)	1.74 (1.30−2.32)
Born in a Nordic country^b^	0.63 (0.32−1.23)	0.76 (0.38−1.52)
Living in a city^c^	1.00 (0.75−1.32)	0.98 (0.73−1.32)
Any university education	1.41 (1.07−1.88)	1.35 (0.97−1.82)
CPR course < 5 years	2.03 (1.54−2.68)	1.60 (1.19−2.16)

Unweighted analysis among non-healthcare professionals reporting self-estimated health good enough to perform CPR, (n = 861). Absolutely yes/yes maybe vs. likely not/absolutely not interested in becoming a smartphone app responder. OR = Odds Ratio. CI = Confidence interval.

a 18−65 vs. > 65 years.

b Nordic country vs. all other countries.

c Cities vs. all other municipalities.

## Discussion

This survey identified potential areas of improvements in general CPR training. Although a majority had participated in a CPR course, only seven percent would have performed the two most important interventions when assessing an unresponsive person with agonal breathing. Attitudes towards becoming a smartphone app responder were promising, particularly among younger people, men and healthcare professionals.

Our first hypothesis, that recent CPR training (< 5 years) would increase the likelihood of correctly identifying the two most important interventions in an agonal breathing case vignette could not be confirmed. Our second hypothesis, that recent CPR training would increase willingness to become a smartphone app responder, was supported.

### Response level in relation to recent CPR training

Only a minority correctly identified the two most important interventions (emergency call and CPR) in the agonal breathing case vignette, and a history of CPR training (< 5 years) did not improve correct identification. Results were similar if CPR training had been performed within one year. This adds to the previous knowledge that agonal breathing presents a major challenge to effective interventions by laypeople in OHCA.[14], [23] Twice as many chose non-effective initial OHCA interventions (Level D). The lack of association between recent CPR training and response quality further demonstrates the complexity of CPR training and knowledge retention. Importantly, this finding indicates that theoretical CPR training is insufficient despite widespread CPR training in society. This is in line with findings by Bakke et al. (2017).^18^

This study indicates a need for more theoretical training with focus on repetition and retention of knowledge, rather than that CPR training is ineffective. CPR is important but is of no use if the need for CPR is not correctly identified. On the other hand, our results show that 70 percent would make the emergency call, enabling emergency medical dispatchers to identify the cardiac arrest. However, agonal breathing may still be a confusing factor for both laypeople and medical dispatchers[14], [23] risking prolonged periods of no-flow. A recent review paper showed that approximately every fourth OHCA remained unrecognised by emergency dispatchers during the emergency call, with large variations between studies.^32^ Using CPR instruction videos with audio files where gasping is highlighted^33^ may be beneficial in order to improve knowledge retention, both in CPR training for laypeople and healthcare professionals.

A possible reason for the relatively poor performance in the case vignette may be that a theoretical case scenario does not fully reflect a real-life cardiac arrest situation and does not capture a potential interaction between bystanders. In a real-life OHCA situation, bystanders may improve their response by interacting with each other.

### Recruitment of smartphone app responders

Smartphone app responder is one of the first responder programs that has been implemented in some regions in Sweden,[7], [8] and seems promising.^8^ A key result in this study was the relatively large proportion interested in becoming smartphone app responders, in a region where it has yet to be implemented. Our survey shows that a majority of the general population has attended a CPR course, and that willingness to intervene is high.[19], [20]

It may be beneficial with targeted recruitment, especially among younger people, men and healthcare professionals. University students could thus potentially be recruited during mandatory CPR training, nursing students and medical students in particular.

### Source of CPR training

Recurrent CPR training in society is a great challenge that requires significant resources. Our study showed that employers are a common source of CPR training.^18^ In our survey, 17 percent quoted an educational institution as a source of CPR training, which is lower than previously reported (27%).^34^ In several countries, e.g. Norway, CPR training is mandatory when obtaining a driver’s license, whereas in Sweden CPR training is optional. In Sweden, 0.3 percent quoted driving school as a source for CPR training, compared to 13% in Norway.^18^

Employers were the main source of CPR training, but do not reach the elderly.[17], [22], [34] OHCA in the elderly typically occur at home, and spouses are the most common group of OHCA bystanders.[22], [35] Reaching the older population may thus be challenging due to a lack of natural contacts, such as school or work.^22^

### CPR knowledge

Good quality CPR achieved through regular hands-on training does not reach its full potential until the need for CPR is identified. A contributing factor for the remaining high mortality after OHCA in Sweden, a country with > 5 million CPR educated citizens,^36^ may be shortcomings in identifying OHCAs. The present survey shows that agonal breathing needs further focus during CPR training in order to improve OHCA recognition and outcomes.^37^ Annual training sessions may not be sufficient for a high quality CPR response, instead a ‘low dose – high frequency model’, as suggested by the ERC guidelines, may be a better strategy[12], [38] Yet, frequent CPR training may be difficult to achieve as shown in this survey, where just 38 percent of healthcare professionals had participated in a CPR course during the last year.

The present survey identified a discrepancy regarding knowledge of AED placement when at work/in school, compared to when at home. Despite increasing numbers of public AEDs, bystander use remains low.^39^ Reasons are probably multifactorial, but suboptimal AED placement in relation to where OHCAs actually occur may be important.^40^ Asking participants in CPR courses about their knowledge about AED placement in their home environment may increase their awareness of them.

The majority of respondents had knowledge of the recommended CPR algorithm; yet 72 percent vastly overestimated survival after OHCA to ‘television levels’.[41], [42] Overestimating OHCA survival may be problematic,^43^ and a more realistic view should be emphasised during CPR courses.

### Strengths and limitations

Data collection through a web-based market company may be biased. First, participants in web panels are more likely to live in major cities and to be better educated. We tried to address this potential web-panel effect by using weighted data. Additionally, panel-members may be more likely to participate in CPR activities, increasing the risk of a selection bias, which needs to be taken into consideration. The risk of recall bias must be considered, as well as the limitation of all data being self-reported, risking that respondents actively seek correct answers on the internet.^44^ In addition, the survey was not formally validated. Furthermore, the difficulties in presenting a real-life simulation in a case vignette should be acknowledged. Finally, attitudes to CPR may have changed due to COVID-19,^45^ and all results in study may not be applicable during a pandemic.

## Conclusions

This study highlights possible areas of improvement in CPR training, which might help identify OHCA and facilitate knowledge retention. The potential to recruit smartphone app responders seems promising in certain groups.

## Funding

The Foundation of Cardiopulmonary resuscitation in Sweden. Lions Research Council, Skåne. The Regional Healthcare system of southern Sweden. None of these funding sources had any influence on the present study.

## Declarations of interest

None.

## CRediT authorship contribution statement

**Cecilia Andréll:** Conceptualization, Methodology, Validation, Formal analysis, Investigation, Resources, Writing - original draft, Writing - review & editing, Visualization, Project administration, Funding acquisition. **Camilla Christensson:** Methodology, Writing - review & editing. **Liselott Rehn:** Methodology, Writing - review & editing. **Hans Friberg:** Conceptualization, Methodology, Software, Validation, Formal analysis, Investigation, Writing - review & editing, Supervision, Project administration. **Josef Dankiewicz:** Conceptualization, Methodology, Validation, Formal analysis, Writing - review & editing, Supervision.
