# Supplementary material for: Knowledge and attitudes to cardiopulmonary resuscitation (CPR)– a cross-sectional population survey in Sweden
Source: Resusc Plus. 2021 Jan 29;5:100071. doi: 10.1016/j.resplu.2020.100071 (PMC8244385; doi:10.1016/j.resplu.2020.100071)
Supplement: Supplementary file 1 [file mmc1.docx]

**Table A1.** Distribution of municipalities in Skåne County and Sweden

**Division Municipality Abbreviated Skåne
 definition**^28^ **County**

**(Sweden)**

**n=**

A1. Large cities. ≥200,000 citizens in

A. Large cities the largest urban area. 1(3)
and municipalities the largest urban area.

near large cities.

A2. Municipalities near ≥40% of the working

large cities. population commute to 8(43)

work in a large city or
 municipality near a

large city.

B. Medium-sized B1. Medium-sized ≥40,000 but <200,000
town and towns. citizens in the largest 2(21)
municipalities near urban area.

medium-sized towns.

B2. Municipalities near ≥40% of the working
 medium-sized towns. population commute to 13(52)
 work in a medium-sized
 town.

B3. Commuting <40% of the working
 municipalities with a population commute to 0(35)

low commuting rate near work in a medium-sized

medium-sized towns town.

C. Smaller towns/ C1. Smaller towns/ ≥15,000 but <40,000

urban areas and urban areas. citizens in the largest 3(29)
rural municipalities. urban area.

C2. Commuting ≥30% commute to or
municipalities near small from a smaller town. 5(52)
towns.

C3. Rural municipalities. <15,000 citizens in the

largest urban area/low 0(40)

commuting pattern.

C4. Rural municipalities Municipalities in rural
with a visitor industry. Areas that fulfil at least 1(15)
 two criteria^1^ for visitor
 industry

*Total number of 33(290)
 municipalities*

Percent (%). ^1^Criteria for visitor industry: number of overnight stays, retail-, restaurant- or hotel turnover per size of population.

**Table A2.** Sociodemographic characteristics stratified by non-healthcare professionals and healthcare professionals.

**Non-healthcare Healthcare
 professionals professionals**

**n=912 n=149**

**Percent Percent**

**If you were born in Sweden**^1^ **which of the following options**

**best describes your background?**

Both my parents were born in Sweden 91.3 92.8

One of my parents was born outside the
Nordic countries 4.7 6.7

Both my parents were born outside the
Nordic countries 3.1 0.5

Prefer not to answer 1.0 -

**Which is your marital status?**

Married, registered partnership or partner 67.6 63.3

Unmarried 22.4 25.7

Divorced 5.1 8.2

Widow/widower 4.9 2.8

**In which type of
accommodation do you live?**

Single-family house/terraced house 49.8 47.6

An apartment that you own 22.0 13.2

Rented apartment 23.8 33.6

Rented room or student apartment/dorm 2.5 0.9

Other 1.9 4.7

**How many people live in your
accommodation, in addition to yourself?**
(Count persons who live permanently or
regularly)

0 18.3 17.6

1 36.7 43.0

2 21.6 11.8

3 12.2 14.0

4 8.5 10.3

5 or more 2.6 3.3

**Who do you share your
accommodation with?**(lives with you during most of the week)
Alone 18.3 17.6

Alone with children 4.1 2.2

Married, cohabiting, partner 45.2 47.9

Married, cohabiting, partner and children 23.5 19.9

Other adults, parents, siblings 8.9 12.3

**What is your monthly
income before tax?**

<10,000 SEK 7.3 6.7

10,000 – 15,000 SEK 7.9 9.2

15,000 – 20,000 SEK 12.0 8.6

20,000 – 25,000 SEK 12.0 17.9

25,000 – 30,000 SEK 13.3 20.1

30,000 – 35,000 SEK 12.5 8.9

35,000 – 40,000 SEK 9.0 4.4

40,000 – 45,000 SEK 3.8 3.5

45,000 – 50,000 SEK 3.0 0.9

> 50,000 SEK 3.4 2.7

Prefer not to answer 15.9 16.9

**How do you consider
your current state of health?**

Very good 23.3 23.7

Good 49.4 45.7

Moderate 22.0 24.6

Bad 3.8 5.1

Very bad 1.3 0.7

Prefer not to answer 0.1 0.2

Weighted analysis with respect to gender, age, municipalities and level of

education. ^1^n=864 non-healthcare professionals and n=131 healthcare professionals.

**Table A3.** Level of response quality among non-healthcare professionals in a case vignette of an out-of-hospital cardiac arrest with agonal breathing.

**Response quality Percent**

**Level A 7.4**

CPR 30:2 + emergency call 5.6

Chest compressions only + emergency call 1.8

**Level B 6.0**

CPR 30:2 2.7

Chest compressions only 0.2

CPR 30:2 + pulse control 1.7

Chest compressions only + pulse control 1.1

CPR 30:2 + open mouth 0.2

CPR 30:2 + stable lateral position 0.1

**Level C 71.0**

Emergency call 15.8

Emergency call + pulse control 27.7

Emergency call + open mouth 2.6

Emergency call + stable lateral position 24.9

**Level D 15.7**

Open mouth (do not start CPR) 0.1

Pulse control 6.1

Stable lateral position 4.1

Open mouth + pulse control 0.4

Open mouth + stable lateral position 1.0

Check for pulse + stable lateral position 3.8

None of the alternatives 0.2

Weighted analysis with respect to gender, age, municipalities and level of

education. Variables do not sum up to 100 percent as percentages are rounded off to one decimal. CPR 30:2 = Cardiopulmonary resuscitation algorithm, 30 chest compressions and mouth-to-mouth ventilation x 2. Response quality: Level A = emergency call and CPR 30:2 or chest compressions only. Level B = CPR 30:2 or chest compressions only, with or without other options (except emergency call). Level C = emergency call with or without other options (except CPR 30:2 or chest compressions only). Level D = all other options, i.e. answers which did not include emergency call nor CPR 30:2 or chest compressions only.

**Table A4.** Level A quality response in a case vignette of out-of-hospital cardiac arrest with agonal breathing among non-healthcare professionals. Logistic regression model with participation in **CPR training course during the last five years.**

**Logistic regression analysis**

**Univariable Multivariable**

**OR (95% CI) OR (95% CI)**

Age 18-65 years^1^ 0.52 (0.32-0.85) 0.51 (0.30-0.87)
Male gender 1.47 (0.90-2.42) 1.52 (0.92-2.52)

Born in a Nordic country^2^ 3.28 (0.44-24.26) 3.07 (0.41-22.84)

Living in a city^3^ 0.92 (0.56-1.50) 0.89 (0.54-1.47)

Any university education 1.18 (0.72-1.95) 1.34 (0.80-2.24)

CPR course < 5 years 0.80 (0.49-1.31) 1.00 (0.58-1.71)

Logistic regression analysis on unweighted data for non-healthcare professionals (n=910) Level A quality response vs. Level B, C and D quality response. OR = Odds Ratio, CI = Confidence interval. ^1^18-65 vs. >65 years. ^2^Nordic country vs. all other countries. ^3^Cities vs. all other municipalities.

**Table A5.** Level A quality response in a case vignette of out-of-hospital cardiac arrest with agonal breathing among non-healthcare professionals. Logistic regression model with participation in **CPR training course during the last year.**

**Logistic regression analysis**

**Univariable Multivariable**

**OR (95% CI) OR (95% CI)**

Age 18-65 years^1^ 0.52 (0.32-0.85) 0.48 (0.29-0.80)
Male gender 1.47 (0.90-2.42) 1.53 (0.93-2.53)

Born in a Nordic country^2^ 3.28 (0.44-24.26) 3.03 (0.41-22.59)

Living in a city^3^ 0.92 (0.56-1.50) 0.89 (0.54-1.47)

Any university education 1.18 (0.72-1.95) 1.34 (0.80-2.23)

CPR course < 1year 1.19 (0.59-2.40) 1.50 (0.72-3.09)

Logistic regression analysis on unweighted data for non-healthcare professionals (n=910) Level A quality response vs. Level B, C and D quality response. OR = Odds Ratio, CI = Confidence interval. ^1^18-65 vs. >65 years. ^2^Nordic country vs. all other countries. ^3^Cities vs. all other municipalities.
