# Supplementary material for: Knowledge and attitudes to cardiopulmonary resuscitation (CPR)– a cross-sectional population survey in Sweden
Source: Resusc Plus. 2021 Jan 29;5:100071. doi: 10.1016/j.resplu.2020.100071 (PMC8244385; doi:10.1016/j.resplu.2020.100071)
Supplement: Supplementary file 2 [file mmc2.docx]

Survey questions translated from Swedish to English:

**Knowledge and attitudes to cardiopulmonary resuscitation (CPR)** *– a cross-sectional population survey in Sweden.*

| **Background questions** |
| --- |

**Q1. What year were you born? [Open, numeric]**

**Q2. Are you a man or a woman?**

1. Man
2. Woman

**Q3. In which country were you born?**

1. Sweden
2. Another Nordic country (Norway, Denmark, Finland or Iceland)
3. The rest of Europe
4. Another country outside Europe
5. Prefer not to answer

**Q4. If you were born in Sweden, which of the following options best describes your background?**

1. Both my parents were born in Sweden
2. One of my parents was born outside the Nordic countries (Sweden, Norway,

Denmark, Finland or Iceland)

1. Both my parents were born outside the Nordic countries (Sweden, Norway, Denmark, Finland or Iceland)

0. Prefer not to answer

**Q5. In which municipality do you live? [Drop down list 1-33]**

1. Bjuv, 2. Bromölla, 3. Burlöv, 4. Båstad, 5. Eslöv, 6. Helsingborg, 7. Hässleholm, 8. Höganäs, 9. Hörby, 10. Höör, 11. Klippan, 12. Kristianstad, 13. Kävlinge, 14. Landskrona, 15. Lomma, 16. Lund, 17. Malmö, 18. Osby, 19. Perstorp, 20. Simrishamn, 21. Sjöbo, 22. Skurup, 23. Staffanstorp, 24. Svalöv, 25. Svedala, 26. Tomelilla, 27. Trelleborg, 28. Vellinge, 29. Ystad, 30. Åstorp, 31. Ängelholm, 32. Örkelljunga, 33. Östra Göinge

**Q6. What is your postal code? [Open, numeric]**

**Q7. Which is your marital status?**

1. Married, registered partnership or partner
2. Unmarried
3. Divorced
4. Widow/widower

**Q8. In which type of accommodation do you live?**

1. Single-family house/terraced house
2. An apartment that you own
3. Rented apartment
4. Rented room or student apartment/dorm
5. Other

**Q9. How many people live in your accommodation, in addition to yourself?** (Count persons who live permanently or regularly). **[Drop down list (0,1, 2, 3, 4, 5, 6, 7, 8, 9, 10 or more)]**

**[Do not ask if Q9=0] [Multi]**

**Q10. Who do you share your accommodation with?** (i.e. lives with you during most of the week). *You can specify several options!*

1. Parents/siblings
2. Husband/wife/cohabiting/partner
3. Other adults
4. Children 0-11 years
5. Children ≥12 years

**Q11. Which is your highest education?** (If you are a student, choose the education You are currently undergoing. Just mark one option!)

1. Pre-secondary grade education (<10 years)
2. ≤2 years of secondary grade education
3. 2 years of secondary grade education/training school
4. 3-4 years of secondary grade education
5. University education, <3 years (less than 180 credits)
6. University education, ≥3 years (more than 180 credits)

**Q12. What is your main occupation right now?**

1. Student
2. Working
3. Unemployed
4. Sick leave
5. Retired

**Q13. What is your monthly income before tax?**

1. <10,000 SEK
2. 10,000 – 15,000 SEK
3. 15,000 – 20,000 SEK
4. 20,000 – 25,000 SEK
5. 25,000 – 30,000 SEK
6. 30,000 – 35,000 SEK
7. 35,000 – 40,000 SEK
8. 40,000 – 45,000 SEK
9. 45,000 – 50,000 SEK
10. > 50,000 SEK
11. Prefer not to answer

**[Multi]**

**Q14. Do you have, or have you had, any of the following professions?** *You can specify several options!*

1. Assistant nurse
2. Nurse
3. Physician
4. None of these options

**Q15. Have you ever called the emergency number ’112’ due to someone else having an acute illness/injury?**

1. Yes
2. No
3. Do not remember

**Q16. How do you consider your current state of health?**

1. Very good
2. Good
3. Moderate
4. Bad
5. Very bad
6. Prefer not to answer

**Q17. Do you have any long-term illness, health issue or disability that would prevent you from performing cardiopulmonary resuscitation (CPR)?**

1. Yes
2. No
3. Prefer not to answer

**Q18. Have you ever been in a situation where there has been a need to perform cardio-pulmonary resuscitation?**

1. Yes
2. No
3. Do not remember

**[Ask if Q18=1]**

**Q19. Did you perform CPR?**

1. Yes, chest compression
2. Yes, chest compression and ventilation
3. Yes, ventilation
4. No
5. Do not remember

**[Ask if Q19=1, 2 or 3]**

**Q20. What was the most important reason for you to perform CPR?**

1. Nobody else had started CPR
2. I felt comfortable to perform CPR
3. Better to try to do something good, even if I felt uncomfortable
4. The medical dispatcher guided me to perform CPR
5. Other reason

**[Ask if Q19=4]**

**Q21. What was the most important reason for you not to perform CPR?**

1. Someone else had already started CPR
2. I was afraid of causing worse harm/do something wrong
3. I felt insecure about how/what to do
4. I felt incapable to act
5. Other reason

**Q22. Have you ever attended a CPR training course?**

1. Yes
2. No
3. Do not remember

**[Ask if Q22=1][Multi]**

**Q23. Who organized the CPR course?** *You can specify several options!*

1. Pre-secondary or secondary grade school
2. Driver’s license education
3. Military service
4. Vocational training
5. Work
6. Red Cross or other volunteer organisation
7. Private company for CPR training
8. Other
9. Do not remember

**[Ask if Q22=1]**

**Q24. How many times have you participated in a CPR training course? [Drop down list (0,1, 2, 3, 4, 5,** ≥**6, Do not remember)]**

**[Ask if Q22=1]**

**Q25. When was the last time you participated in a CPR training course?**

1. < 1 year ago
2. 1-5 years ago
3. > 5 years ago
4. Do not remember

**[Ask if Q22=1]**

**Q26. Have you, after participating in a CPR training course, ever been in a situation (while at work or in your private life) where there has been a need for CPR?**

1. Yes
2. No
3. Do not know

**[Ask if Q26=1]**

**Q27. Think of the latest CPR situation that you encountered, to which extent do you agree with the following statement: The CPR training course made you more prepared and ready to act in the situation?**

1. Totally agree
2. Partly agree
3. Disagree
4. Do not know

**[Ask if Q27=2 or 3] [Multi]**

**Q28. What did you consider to be missing in the CPR training course?** *You can specify several options!*

1. It has been a long time since I attended the CPR course
2. The course was to intense and brief
3. Too little focus and time for practical training
4. Too big training group
5. Other reason **[Open]**

| **Questions about cardiopulmonary resuscitation** |
| --- |

**Q29. Which statement is correct regarding chest compression and ventilation in CPR for an adult who has collapsed, is unresponsive, and isn’t breathing?**

1. CPR is performed by repeating 15 chest compressions followed by 2 mouth-to-mouth ventilation (15:2, 15:2 …)
2. CPR is performed by repeating 30 chest compressions followed by 2 mouth-to-mouth ventilation (30:2, 30:2 …)
3. CPR is performed by repeating 45 chest compressions followed by 2 mouth-to-mouth ventilation (45:2, 45:2 …)

**Q30. How many percent of individuals affected by out-of-hospital cardiac arrest in Sweden survive for at least 30 days?**

1. 5%
2. 10%
3. 30%
4. 50%

**Q31. How many percent can survive an out-of-hospital cardiac arrest if defibrillation with an automated external defibrillator (AED) occurs within three minutes?**

1. 10%
2. 30%
3. 70%

**Q32. Do you know what the sign looks like that symbolizes the placement of an AED?**

1. Yes
2. No

**Q33. Are you familiar with the location of the nearest AED when you are at home?**

1. Yes
2. No

**[Ask if Q12=1 or 2]**

**Q34. Are you familiar with the location of the nearest AED when you are at work/school?**

1. Yes
2. No

| **Case** |
| --- |

**Q35. You are passing by a person lying on the ground. The person does not react when you shake him/her and he/she is not breathing normally, but snoring with slow breaths. What would you do?** *2 choices maximum!*

1. 30 chest compressions and 2 mouth-to-mouth ventilations, CPR 30:2, 30:2 …
2. Open the mouth, do not start CPR
3. Check if he/she has pulse
4. Place him/her in stable lateral position (previously termed recovering position)
5. Call the emergency number ‘112’
6. Chest compressions (continuously) without mouth-to-mouth ventilation
7. None of the alternatives above

| **At last** |
| --- |

**[Show intro and Q36 on same page]**

**[Intro] *A cardiac arrest responder app*** makes it possible for laypeople to be dispatched (by his/her smartphone) to the site of a suspected out-of-hospital cardiac arrest. The purpose is to shorten time from cardiac arrest to start of CPR start – pending an ambulance response.

A message is dispatched via an application to the cardiac arrest responder’s smartphone. ‘Cardiac arrest layperson responders’ has been implemented in Stockholm and in part of the regions of Västra Götaland, Sörmland, Västmanland. It is soon about to be introduced in other regions in Sweden.

**Q36. If a cardiac arrest responder app is implemented in Skåne, would you be interested in becoming a “cardiac arrest layperson responder”?**

1. Absolutely yes
2. Yes, maybe
3. Likely not
4. Absolutely not

0. Prefer not to answer

**[Ask if Q36=1 or 2]**

**Q37. What is the main reason you answered ‘absolutely yes or maybe’?**

1. I see it as a natural thing to help people in need
2. I feel comfortable to perform CPR and I feel that I can contribute
3. I think it is better to try than to do nothing at all
4. It gives me a reason to participate in a CPR training/retraining course
5. Other reason **[open]**

**[Ask if Q36=3 or 4]**

**Q38. What is the main reason you answered ‘likely not or absolutely not’?**

1. Concerns about data privacy (positioning) in case of a cardiac arrest in my vicinity
2. I am afraid of being infected with a disease
3. I do not want to perform mouth-to-mouth ventilation to a stranger
4. I am afraid of doing something wrong and aggravate the damage
5. I am afraid of being alone in a CPR-situation.
6. I feel that my CPR skills are lacking
7. Other reason **[open]**

**---END---**
